# 手术切除的N2期非小细胞肺癌预后影响因素探究

**DOI:** 10.3779/j.issn.1009-3419.2020.01.03

**Published:** 2020-01-20

**Authors:** 振宇 杨, 诗友 韦, 粱 夏, 伦旭 刘

**Affiliations:** 610041 成都，四川大学华西医院胸外科 Department of Thoracic Surgery, West China Hospital, Sichuan University, Chengdu 610041, China

**Keywords:** 肺肿瘤, TNM分期, 预后, Lung neoplasms, TNM stage, Prognosis

## Abstract

**背景与目的:**

N2期非小细胞肺癌患者预后异质性很强，本研究旨在探索其预后相关因素。

**方法:**

纳入四川大学华西医院胸外科2007年1月-2016年12月间行根治性切除的N2期患者，使用*Cox*模型比较临床病理学特征与总体生存率之间的关系，使用*Kaplan-Meier*法绘制生存曲线，并且根据淋巴结转移情况进行亚组分析。

**结果:**

总共纳入773例N2期患者，中位随访时间57.2个月，5年总体生存率为34.8%。肿瘤原发灶-淋巴结-远处转移（tumor-node-metastasis, TNM）分期、多站淋巴结转移、跳跃性转移、脉管侵犯、辅助化疗为预后相关的独立风险因素。T1-3期患者具有相似的预后，T4期患者预后明显更差。单站跳跃性转移的患者预后最好，5年生存率达48.9%。

**结论:**

N2患者中T4期预后差。在将来的分期中应结合淋巴结转移站数和跳跃性转移进行更精细的N分期划分。

## Abstract (en)

**Background and objective:**

Patients with N2 stage non-small cell lung cancer have prognostic heterogeneity, and this study attempted to explore the prognostic factors among those patients.

**Methods:**

Patients with N2 stage undergoing radical resection in Department of Thoracic Surgery, West China hospital, Sichuan University between January 2007 and December 2016 were included. *Cox* proportional hazard regression model was used to explore the prognostic value of clinicopathological features. Survival curves were plotted by *Kaplan-Meier* method. Subgroup analyses considering the situation of lymph node involvement were performed.

**Results:**

In total, 773 patients were included. The median follow-up time was 57.2 months, and the 5-year overall survival rate was 34.8%. Tumor-node-metastasis (TNM) stage, number of involved lymph node stations, skip metastasis, lymphatic or vascular invasion and adjuvant chemotherapy were independent prognostic factors. The patients with stage T1-3 had similar prognosis, while the patients with stage T4 had worse survival. In addition, the patients with single station involvement and skip metastasis had the best prognosis with a 5-years overall survival rate of 48.9%.

**Conclusion:**

T4 stage patients had worse survival in N2 group. To get a more precisely stratification, skip metastasis and number of involved lymph node stations should be considered in future N stage classification.

肺癌的发病率和病死率高，在全球范围内带来极大的疾病负担^[1]^。肺癌的5年生存率仅有17.7%，在肿瘤死亡中占据主要部分^[2]^。国际肺癌研究协会（International Association for the Study of Lung Cancer, IASLC）构建的非小细胞肺癌（non-small cell lung cancer, NSCLC）肿瘤原发灶-淋巴结-远处转移（tumor-lymph node-metastasis, TNM）分期系统对于NSCLC患者的治疗指导及预后判断意义重大^[2, 3]^。N2M0期NSCLC的5年生存率在25%-46%^[2-4]^，不同患者间预后差异较大。目前NSCLC的常见预后临床因素包含年龄、性别、吸烟史、TNM分期以及辅助治疗等^[5]^，近年来有学者提出淋巴结转移站数以及是否跳跃性转移是N2患者的重要预后特征^[6-8]^。探索影响N2期预后因素有助于进行更精准的患者分层并指导治疗。本研究基于四川大学华西医院胸外科2007年1月-2016年12月收治的行根治性手术切除的N2期NSCLC患者的生存资料及相关临床病理学特征的数据来探索N2期NSCLC患者预后差异的主要因素。

## 1 资料与方法

### 1.1 一般资料

回顾性分析四川大学华西医院胸外科、中国西部肺癌数据库中前瞻性收集的2007年1月-2016年12月肺癌患者随访数据。中国西部肺癌数据库自动从医院病历记录中提取相关数据，该数据库由2名全职科研助理维护，由2名教授监管。本研究通过华西医院伦理审查委员会审查（伦理号2019908）。本研究纳入手术切除的N2期NSCLC患者，纳入标准：术后病理检查证实为N2期NSCLC，手术切除为根治性切除且行系统性淋巴结清扫的病人（参照欧洲胸外科医师协会指南：至少应评估3组N1站淋巴结和包括隆突下淋巴结在内的3组以上的纵隔淋巴结）。排除标准：接受术前新辅助治疗（含化疗、放疗、靶向治疗），既往有其他肿瘤病史，术后病理发现合并小细胞肺癌成分，有远处转移，术后4周内死亡的患者。

### 1.2 研究方法

患者的病理分期依据IASLC提出的第八版肺癌TNM分期系统，其中N分期判定依据IASLC淋巴结分区图谱。患者的临床特征、肿瘤特征、手术记录、生存信息均从西部肺癌数据库中提取。本研究结局指标为总生存期（overall survival, OS）以及无疾病生存期（disease-free survival, DFS）。跳跃性淋巴结转移为N1站淋巴结病理检测阴性而N2站淋巴结阳性。根据淋巴结转移站数和是否跳跃性转移，结合第八版分期的提议，将N2期患者分为单站跳跃性转移（N2a1）、单站非跳跃性转移（N2a2）、多站跳跃性转移（N2b1）、多站非跳跃性转移（N2b2）。所有患者均在术后1个月于门诊复查，术后2年每3个月-6个月复查，3年-5年每6个月复查，此后每年规律复查。部分无法进行门诊评估的病人行电话随访。

### 1.3 统计学方法

使用R软件（版本3.5.1）进行统计学分析。使用单因素*Cox*比例风险回归模型评估临床病理特征及手术方式与OS的关系。在单因素中P值小于0.1的因素以及临床上认为与预后相关的因素纳入多因素*Cox*比例风险回归模型中进行多因素分析，以风险比（hazard ratio, HR）和95%置信区间（confidence interval, CI）作为统计学指标。OS和DFS通过*Kaplan-Meier*方法进行计算以及*Log-rank*法进行组间比较。所有检验均采用双侧检验，*P* < 0.05视为有统计学差异。本研究使用R包“survival”及“survminer”分别进行生存分析和绘制生存曲线。

## 2 结果

### 2.1 纳入人群临床及病理特征

2007年1月-2016年12月期间在四川大学华西医院胸外科接受手术治疗的肺癌患者共6471例，符合纳入与排除标准的共773例。患者具体临床病理资料见表 1。纳入人群的年龄中位值为57岁（四分位间距为50岁-63岁），其中472例为男性，301例为女性；有吸烟史377例，无吸烟史为396例；Ⅲa期为546例（T1期为58例，T2期为488例）；Ⅲb期为227例（T3期为137例，T4期为90例）；跳跃性转移共233例（N2a1为153例，N2b1为80例），非跳跃性转移540例（N2a2为236例，N2b2为304例）；病理类型上，514例为腺癌，259例为非腺癌，有脉管侵犯的38例；在辅助治疗方面，548例患者接受辅助化疗，175例患者接受放疗，65例患者接受靶向治疗（化疗+放疗163例，化疗+靶向22例，放疗+靶向3例，化疗+放疗+靶向7例）。中位随访时间为57.2个月（四分位间距为40.4个月-76.2个月）。

### 2.2 N2期NSCLC预后因素分析

N2期患者整体5年OS为34.8%。采用*Cox*比例风险回归模型对纳入人群的OS及临床病理学特征进行分析（表 1）。单因素分析显示，女性、无吸烟史、Ⅲa期（即T1、T2期）、N2淋巴结跳跃性转移、N2单站转移、无脉管侵犯、辅助治疗与较好的OS相关；肿瘤位置、手术方式、手术切除范围、病理类型与N2期患者的预后无关。在多因素*Cox*分析中纳入与OS相关的指标，结果显示，Ⅲa期（HR=1.35, 95%CI: 1.11-1.65, *P*=0.003），非跳跃性转移（HR=1.39, 95%CI: 1.13-1.73, *P*=0.002），N2多站淋巴结转移（HR=1.51, 95%CI: 1.25-1.82, *P* < 0.001），有脉管侵犯（HR=1.59, 95%CI: 1.04-2.43, *P*=0.034），辅助化疗（HR=0.65, 95%CI: 0.53-0.81, *P*=0.034），均为预后相关的独立风险因素。

**1:** 患者特征和单因素多因素*Cox*分析 Patients characteristics and *Cox* proportional hazard regression analysis

Index	*n* (%)	Univariate analysis				Multivariate analysis		
		HR	95%CI	*P*		HR	95%CI	*P*
Age (Median, IQR)	57.0% (50.0%-63.0%)	1.01	1-1.02	0.251		1	0.99-1.01	0.561
Gender						0.86	0.65-1.15	0.317
Male	472 (61.1%)	Ref						
Female	301 (38.9%)	0.8	0.66-0.97	0.023				
Smoking history						1.15	0.87-1.52	0.321
No	377 (48.8%)	Ref						
Yes	396 (51.2%)	1.24	1.03-1.48	0.023				
Location								
Left	368 (47.6%)	Ref						
Right	405 (52.4%)	1.05	0.88-1.26	0.567				
Surgical method								
Open surgery	379 (49.0%)	Ref						
VATS	394 (51.0%)	0.88	0.74-1.06	0.187				
Extent of pulmonary resection								
Sublobar/wedge resection	11 (1.4%)	Ref						
Lobectomy/Bilobectomy	722 (93.4%)	1.00	0.45-2.23	0.996				
Pneumonectomy	40 (5.2%)	1.31	0.54-3.2	0.546				
Tumor size (mean, cm)	4.0 (±1.9)	1.08	1.03-1.13	0.001				
AJCC stage 8th edition						1.35	1.11-1.65	0.003
Ⅲa	546 (70.6%)	Ref						
Ⅲb	227 (29.4%)	1.38	1.14-1.68	0.001				
T stage								
T1	58 (7.5%)	Ref						
T2	488 (63.1%)	1.11	0.77-1.6	0.587				
T3	137 (17.7%)	1.27	0.84-1.9	0.252				
T4	90 (11.7%)	1.99	1.31-3.02	0.001				
Lymph node								
Skip	233 (30.1%)	Ref						
Non-skip	540 (69.9%)	1.45	1.18-1.79	< 0.001		1.39	1.13-1.73	0.002
N2 one station	389 (50.3%)	Ref						
N2 multiple stations	384 (49.7%)	1.62	1.36-1.94	< 0.001		1.51	1.25-1.82	< 0.001
Histological type								
Adenocarcinoma	514 (66.5%)	Ref						
Non-adenocarcinoma	259 (33.5%)	1.02	0.84-1.23	0.862				
Lymphatic/vascular invasion						1.59	1.04-2.43	0.034
No	735 (95.1%)	Ref						
Yes	38 (4.9%)	1.5	0.98-2.28	0.061				
Adjuvant treatment^*^								
Chemotherapy	548 (70.9%)	0.60	0.49-0.73	< 0.001		0.65	0.53-0.81	< 0.001
Radiotherapy	175 (22.6%)	0.63	0.48-0.81	< 0.001		0.82	0.64-1.05	0.109
Targeted therapy	65 (8.4%)	0.57	0.4-0.82	0.003		0.73	0.52-1.04	0.080
^*^overlaps exist between different treatment groups; VATS: video-assisted thoracic surgery; AJCC: American Joint Committee on Cancer; HR: hazard ratios; CI: confidence interval.								

### 2.3 T分期及N2期淋巴结转移情况

T1、T2、T3、T4期患者5年OS分别为35.7%、37.7%、35.2%、19.6%，5年DFS分别为19.5%、25.8%、23.9%、12.6%（图 1）。Ⅲa（T1、T2）期较Ⅲb期（T3、T4）预后较好，同时对比T4期患者，T1+T2+T3期预后更好，统计学差异更明显（HR=1.72, 95%CI: 1.33-2.24, *P* < 0.001）。根据淋巴结转移站数和是否跳跃性转移，将N2期NSCLC患者划分为N2a1、N2a2、N2b1、N2b2四个亚组，比较组间生存差异。在四个亚组中，5年OS分别为48.9%、40.4%、26.7%、24.4%，5年DFS分别为38.4%、27.6%、20.9%、12.0%（图 2）。利用多因素*Cox*模型矫正性别、分期、吸烟史等危险因素后（表 2），N2a2（OS: HR =1.49, 95%CI: 1.12-1.99, *P*=0.007; DFS: HR=1.43, 95%CI: 1.09-1.87, *P*=0.011）、N2b1（OS: HR=1.68, 95%CI: 1.16-2.44, *P*=0.006; DFS: HR=1.55, 95%CI: 1.18-2.43, *P*=0.004）、N2b2（OS: HR=2.16, 95%CI: 1.65-2.84, *P* < 0.001; DFS: HR=2.23, 95%CI: 1.72-2.90, *P* < 0.001）预后较N2a1更差，N2b2较N2a2（OS: HR=1.43, 95%CI: 1.15-1.79, *P*=0.001; DFS: HR=1.51, 95%CI: 1.21-1.87, *P* < 0.001）预后更差，但在N2b2和N2b1组间生存情况无统计学差异（OS: HR=1.25, 95%CI: 0.90-1.72, *P*=0.179; DFS: HR=1.27, 95%CI: 0.93-1.73, *P*=0.138）。

**1:**
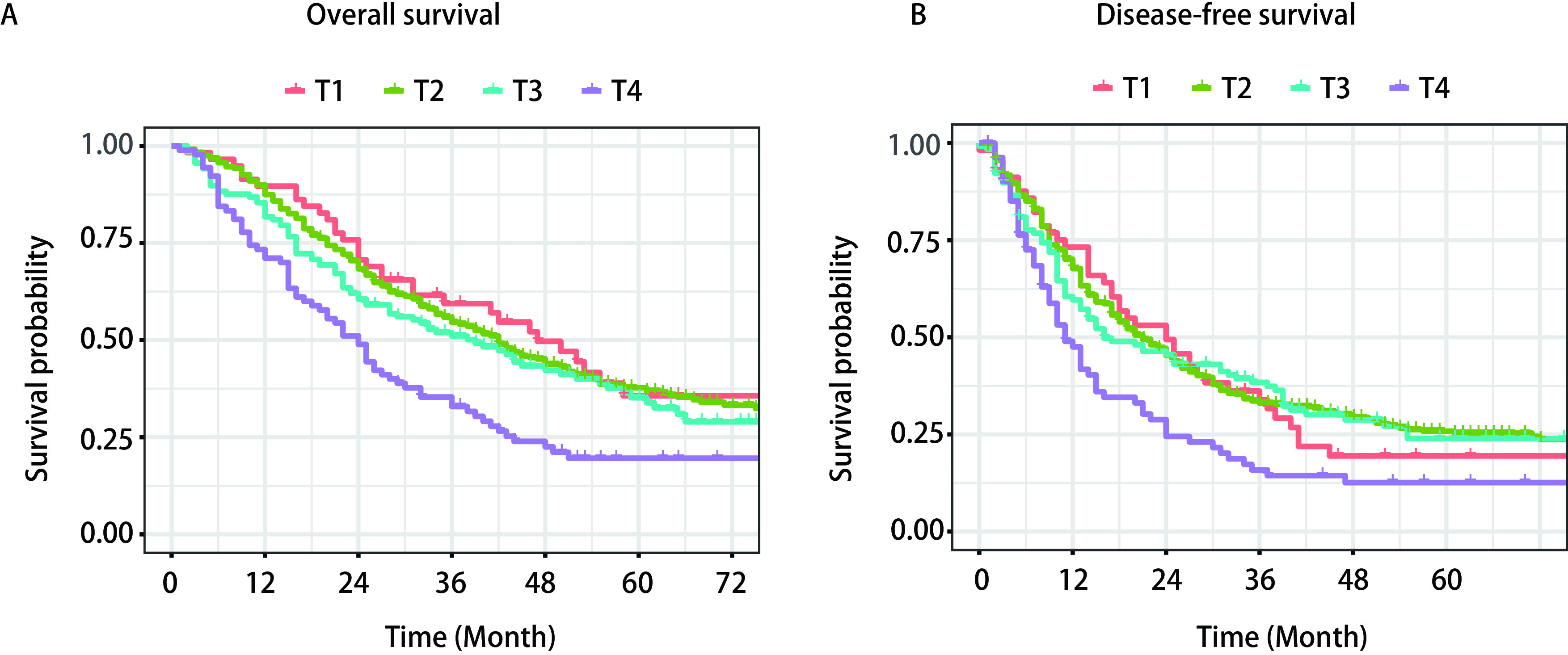
T分期与总体生存率（A）和无病生存率（B）生存曲线 Survival curves of T stage in overall survival (A) and disease-free survival (B)

**2:**
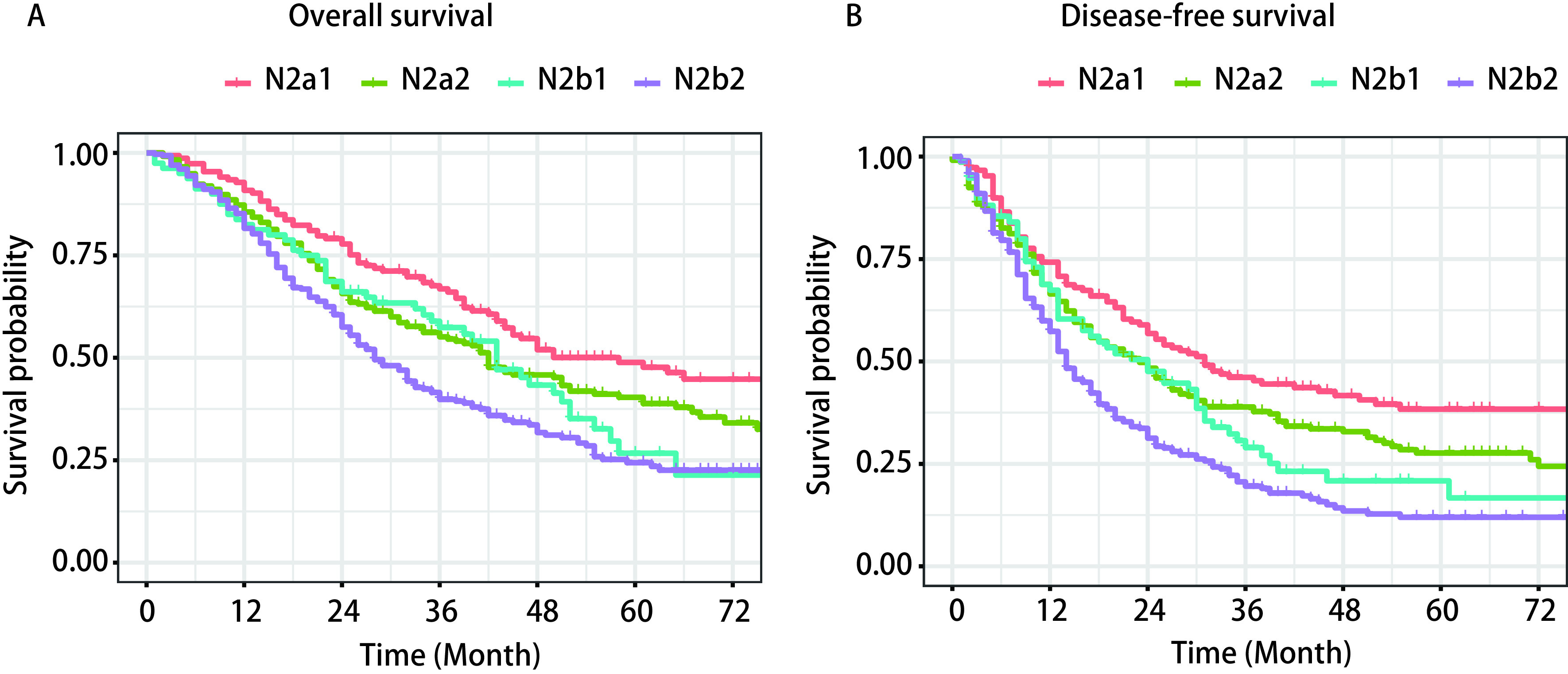
淋巴结转移情况与总体生存率（A）和无病生存率（B）的生存曲线及多因素*Cox*分析结果 Survival cures and multivariate *Cox* model between of subgroup analyses considering lymph node involvement in overall survival (A) and disease-free survival (B)

**2:** N分期各亚组间多因素*Cox*分析 Multivariate *Cox* proportional hazard regression analysis in subdivided N stage

	Overall survival				Disease-free survival		
	HR	95%CI	*P*		HR	95%CI	*P*
N2a2 *vs* N2a1	1.49	1.12-1.99	0.007		1.43	1.09-1.87	0.011
N2b1 *vs* N2a1	1.68	1.16-2.44	0.006		1.55	1.18-2.43	0.004
N2b2 *vs* N2a1	2.16	1.65-2.84	< 0.001		2.23	1.72-2.90	< 0.001
N2b1 *vs* N2a2	1.13	0.81-1.58	0.454		1.23	0.89-1.69	0.207
N2b2 *vs* N2a2	1.43	1.15-1.79	0.001		1.51	1.21-1.87	< 0.001
N2b2 *vs* N2b1	1.25	0.90-1.72	0.179		1.27	0.93-1.73	0.13

## 3 讨论

对本研究机构的773例N2期NSCLC患者进行回顾性分析，N2期患者整体5年生存率为34.8%，影响N2期预后的主要因素包括：疾病分期、是否跳跃性转移、淋巴结转移站数、是否脉管侵犯、是否行辅助化疗。

在肿瘤的TNM分期上，目前N2期患者根据T分期划分为Ⅲa期（T1、T2）与Ⅲb期（T3、T4），Ⅲb期患者的预后次于Ⅲa期（HR=1.35）。在单因素*Cox*分析中，观察到T1、T2、T3期的单因素比较无差异（表 1）并且5年生存率接近，而T4期差异明显（HR=1.99, 95%CI: 1.31-3.02, *P*=0.001），因此我们对T分期进行探索性研究：将T分期分为T1+T2+T3期对比T4期，纳入多因素*Cox*分析中进行比较，结果显示，这种划分方式较第八版分期推荐的划分方式差异更为显著。对于N2期转移的患者，肿瘤大小及局部侵犯的特征对患者的预后影响较小，当肿瘤达到T4期时对患者的预后影响明显。在纳入的人群中，T1期患者较少，仅58例，因此未能按照更细的T分期进行比较^[9]^。此外，本研究中有38例患者出现脉管侵犯，该类患者预后明显次于无脉管侵犯患者，此结果与前人的研究一致，脉管侵犯是预后不良的重要因素^[10-12]^。

对于淋巴结分期，目前IASLC第八版分期中仍延用第七版N分期依据，以受侵犯淋巴结的区域作为N分期的标准，但在八版分期中对淋巴结分期提议考虑淋巴结转移的站数和是否跳跃性转移^[13]^。此前有多项研究发现，跳跃性转移的患者预后较好^[7, 8, 14, 15]^，多站N2转移的患者预后更差^[6]^。在本研究中，这两项因素是N2期NSCLC患者的独立预后因素。当肿瘤侵犯多站N2淋巴结时，患者的整体死亡风险增加51%，在非跳跃性转移时患者整体死亡风险增加39%。由此可见，在手术中更加系统的清扫淋巴结，获取更准确的淋巴结转移情况对于评估患者的预后有重要价值。为了进一步探索上述两项因素对患者预后的影响，我们进行了亚组分析，发现N2a1期患者5年OS为48.9%，5年DFS为38.4%，其预后明显优于其他三组（N2a2期、N2b1期、N2b2期），统计学差异均显著。但在N2期多站转移中，N2b2期与N2b1期没有明显差异。此前来自韩国的学者研究报道提出，N2a1期患者的预后情况及临床特征较N1期患者更为接近，对于N2期患者或可划分为N2a1期、N2a2期以及N2b期三组^[16]^。在我们的结果中，尽管N2b1期（HR=1.23）、N2b2期（HR=1.51）患者预后较N2a2期患者更差，但N2a2期患者与N2b1期并无差异，此划分方式有待进一步的验证。

此外，在辅助治疗中，美国综合癌症网络（National Comprehensive Cancer Network, NCCN）推荐对于N2期NSCLC患者使用术后化疗^[17]^，化疗的主要方案以铂类药物为主辅以长春花碱或培美曲塞等^[18, 19]^。在本研究中同样证明术后化疗能够有效提高患者的生存率（HR=0.65, 95%CI: 0.53-0.81, *P*=0.034）。在一项基于大规模人群的研究中，对N2期患者使用辅助放疗能够提高患者预后^[20]^，但我们的数据显示，虽然放疗是一项预后因素（HR=0.63, 95%CI: 0.48-0.81, *P* < 0.001），但在矫正其他预后相关混杂因素后无统计学差异。靶向治疗亦是肺癌治疗的重要手段，对比无辅助治疗患者，靶向治疗可显著提高预后（HR=0.57, 95%CI: 0.4-0.82, *P*=0.003），但在多因素分析中无统计学差异，此结果可能是研究纳入的人数有限所导致。新辅助治疗亦是N2期患者的重要治疗方式，但是为获得真实的淋巴结分期，本研究未纳入该类患者。因多数临床分期N2的患者推荐新辅助治疗，本研究中78.9%（610/773）的患者在术前未预测到出现N2淋巴结转移。

总体来讲，我们通过对手术切除的N2期NSCLC患者回顾性分析发现，N2期NSCLC患者预后异质性的来源包括肿瘤T分期、脉管侵犯情况、淋巴结转移情况以及是否化疗。通过亚组分析发现，T3N2期患者的预后与T1-2N2期患者接近。跳跃性转移和淋巴结转移站数在N2期患者中预后价值明显，N2单站跳跃性转移患者5年OS接近50%，此两项因素在未来的分期修订中应纳入考虑。本研究属于单中心回顾性研究，未纳入新辅助治疗患者，更进一步的结论需要多中心的前瞻性研究来证实。

## Author contributions

Yang ZY, Wei SY and Liu LX conceived and designed the study. Wei SY, Xia L collected the data. Yang ZY analyzed the data and contributed analysis tools. Xia L and Liu LX provided critical inputs on design, analysis, and interpretation of the study. All the authors had access to the data. All authors read and approved the final manuscript as submitted.
